# Type XXII Collagen Complements Fibrillar Collagens in the Serological Assessment of Tumor Fibrosis and the Outcome in Pancreatic Cancer

**DOI:** 10.3390/cells11233763

**Published:** 2022-11-24

**Authors:** Emilie A. Madsen, Jeppe Thorlacius-Ussing, Neel I. Nissen, Christina Jensen, Inna M. Chen, Julia S. Johansen, Hadi M. H. Diab, Lars N. Jørgensen, Carsten P. Hansen, Morten A. Karsdal, Nicholas Willumsen

**Affiliations:** 1 Biomarkers and Research, Nordic Bioscience A/S, Herlev Hovedgade 207, 2730 Herlev, Denmark; 2 Department of Biomedical Sciences, University of Copenhagen, 2200 Copenhagen, Denmark; 3 Department of Oncology, Copenhagen University Hospital—Herlev and Gentofte, 2730 Herlev, Denmark; 4 Department of Medicine, Copenhagen University Hospital—Herlev and Gentofte, 2730 Herlev, Denmark; 5 Department of Clinical Medicine, Faculty of Health and Medical Sciences, University of Copenhagen, 2200 Copenhagen, Denmark; 6 Digestive Disease Center, Bispebjerg Hospital, University of Copenhagen, 2400 Copenhagen, Denmark; 7 Department of Surgery, Rigshospitalet, University of Copenhagen, 2100 Copenhagen, Denmark

**Keywords:** collagens, ECM, non-invasive biomarker, PDAC, tumor fibrosis, type XXII collagen

## Abstract

Circulating fragments of type III collagen, measured by PRO-C3, has shown promising results as a tumor fibrosis biomarker. However, the fibrotic tumor microenvironment consists of many other collagens with diverse functions and unexplored biomarker potential. One example hereof is type XXII collagen (COL22). In this study, we investigated the biomarker potential of COL22 by measuring this in serum. An ELISA, named PRO-C22, was developed and measured in two serum cohorts consisting of patients with various solid tumors (n = 220) and healthy subjects (n = 33) (Cohort 1), and patients with pancreatic ductal adenocarcinoma (PDAC) (n = 34), and healthy subjects (n = 20) (Cohort 2). In Cohort 1, PRO-C22 was elevated in the serum from patients with solid tumors, compared to healthy subjects (*p* < 0.01 to *p* < 0.0001), and the diagnostic accuracy (AUROC) ranged from 0.87 to 0.98, *p* < 0.0001. In Cohort 2, the high levels of PRO-C22, in patients with PDAC, were predictive of a worse overall survival (HR = 4.52, 95% CI 1.90–10.7, *p* = 0.0006) and this remained significant after adjusting for PRO-C3 (HR = 4.27, 95% CI 1.24–10.4, *p* = 0.0013). In conclusion, PRO-C22 has diagnostic biomarker potential in various solid tumor types and prognostic biomarker potential in PDAC. Furthermore, PRO-C22 complemented PRO-C3 in predicting mortality, suggesting an additive prognostic value when quantifying different collagens.

## 1. Introduction

Globally, cancer is one of the most common causes of death [1] and by 2040, more than 29 million new cancer cases are expected worldwide [2]. Cancer presents heterogeneously and each cancer type has a different prevalence and mortality rate. Especially, pancreatic cancer deaths are increasing worldwide: With a 5-year overall survival (OS) rate of 11% [3] and high incidence rates, pancreatic cancer is projected to be the third leading cause of cancer-related death by 2025 [4,5]. This emphasizes the need for novel biomarker tools to aid in the management of pancreatic cancer.

The tumor microenvironment (TME) plays an important role in various aspects of the development and progression of pancreatic cancer [6]. Apart from affecting cancer cell migration, invasion, metastasis, and angiogenesis, the TME modulates the response to anti-cancer treatments [7,8,9,10,11,12]. The TME, therefore, holds the potential as a source of novel biomarkers and therapies. The TME comprises cancer cells, immune cells, stromal cells, and extracellular matrix (ECM). The ECM makes up the large noncellular component of tissues and aids in the organization and stability of tissues [13]. In healthy tissue, the ECM homeostasis is kept in balance by a constant remodeling of the ECM components. This balance is lost in solid tumors, where excessive remodeling of the ECM occurs [14]. This includes the increased proteolytic activity and the degradation of basement membrane components associated with migration and invasion. In addition, it includes a massive deposition and cross-linking of major fibrillar collagens, the main constituents of the interstitial matrix (IM). This process commonly involves collagen types I (COL1) and III (COL3) surrounding the tumor cells and is mediated by the myofibroblastic cancer-associated fibroblasts (CAFs), resulting in a dense and collagen-rich TME, termed tumor fibrosis [15,16]. This excessive accumulation of major fibrillar collagens has been associated with poor OS [17,18,19,20,21]. The representative ECM fragments, released into the circulation, can be non-invasively detected in a liquid biopsy. These fragments reflect pathological processes including tumor fibrosis activity and can be used to describe the dynamics of ECM remodeling. The information provided by these types of biomarkers go beyond what is typically found in omics- or array-based studies, because they reflect the proteolytic activity not detectable in intact, full-length proteins [16,22,23,24].

The fibrillar collagens COL1 and COL3 are some of the most studied collagens in cancer, however 28 collagens have been identified, and many remain unexplored. The different collagens have unique properties and are central components of the ECM [25,26,27]. A lesser-known subgroup of collagens is the fibril-associated collagens with interrupted triple helices (FACITs) [28]. FACITs are essential for ensuring the stability and integrity of the collagen network in the ECM [29,30]. Most FACITs exert their primary function during the tissue development [31,32,33,34,35]. The FACITs include collagen types IX (COL9), XII (COL12), XIV (COL14), XVI (COL16), XIX (COL19), XX (COL20), XXI (COL21), and XXII (COL22) [29]. COL9 is primarily associated with cartilage; COL12 with invasion in cancer; and COL14 with regulating fibrillogenesis, interstitial collagen network, and late-stage fibrosis [25,29,36,37,38,39]. The serological assessment of COL16, COL19, and COL20, has shown potential as cancer biomarkers [40,41,42]. Specifically, the circulating levels of COL19 and COL20 are significantly elevated in serum from patients with cancer, compared to healthy subjects [41,42]. COL22 is an understudied collagen even among the FACITs, therefore the function is not fully understood. However, the gene encoding COL22, COL22A1, is associated with the matrix formation and remodeling in bone and cartilage, as well as cardiac fibrosis [43,44,45,46]. Furthermore, the mRNA levels of COL22 have shown to have a prognostic value in patients with head and neck cancer [47]. These data indicate that COL22 is involved in tumor fibrosis and could have the potential as a biomarker in cancers.

This study aimed to establish a robust ELISA-based biomarker assay to assess the serological protein levels of COL22 and to evaluate the biological relevance of the biomarker in patients with cancer.

## 2. Materials and Methods

### 2.1. Generation of Monoclonal Antibodies Targeting the C-Terminal of Type XXII Collagen

A ten amino acid (AA) peptide ^1616^AARPGNVKGP^1626^ corresponding to the C-terminus of type XXII collagen (UniprotKB: Q8NFW1) was purchased from Genscript (Piscataway, NJ, USA) and used for immunization. The peptide used for immunization was generated by covalently cross-linking the target peptide to the keyhole limpet hemocyanin (KLH) carrier protein using sulfosuccinimidyl 4-(N-maleimidomethyl) cyclohexane-1-carboxylate, SMCC (Thermo Scientific, Waltham, MA, USA, cat. no. 22322). Six-week-old Balb/C female mice were subcutaneously injected with emulsified KLH-CGG-conjugated immunogenic peptide (KLH-CGG-^1616^AARPGNVKGP^1626^). Glycine and cysteine residues were added at the N-terminal end to ensure the correct linking of the carrier protein. Immunizations using 200 µL emulsified antigen containing 100 µg immunogenic peptide mixed with Sigma adjuvant system (Sigma cat. No. S6322) were performed at 2-week intervals until stable sera titer levels were reached. The mouse with the highest titer was rested for four weeks and was then boosted with 100 µg of immunogenic peptide in 100 µL 0.9% NaCl solution intravenously and terminated. The hybridoma cells were produced by fusing the spleen cells with SP2/0 myeloma cells, as previously described [48]. The resultant hybridoma cells were then cultured in 96-well microtiter plates, and subcloning was carried out while limiting the dilution of the cells. The selected monoclonal cells were seeded in 24-well plates and then expanded in T25, then T75, and finally in T150 flasks before collecting the supernatants. The 500 mL supernatant was collected and purified using protein-G-columns, according to the manufacturer’s instructions (GE Healthcare Life Sciences, Little Chalfont, UK, cat. no. 17-0404-01).

### 2.2. PRO-C22 ELISA Protocol

A 96-well streptavidin-coated ELISA plate was coated with 100 µL/well of 20 ng/mL biotinylated ^1616^AARPGNVKGP^1626^ peptide (Biotin-^1616^AARPGNVKGP^1626^) dissolved in an assay buffer (25 mM TBS-BTB, 2 g/L NaCl, pH 8.0) and incubated for 30 min at 20 °C, shaking at 300 rounds per minute (rpm). Following five washing cycles with a washing buffer (25 mM Tris, 50 mM NaCl, pH 7.2), 20 µL/well of the serum sample (diluted 1:2, in assay buffer) was added in duplicates, followed by 100 µL/well of 35 ng/mL horseradish peroxidase-labeled (HRP-labeled), using a peroxidase labeling kit (Roche Diagnostics GmbH, Mannheim, Germany, cat. no. 11829696001) monoclonal antibody (HRP-mAb) in an assay buffer. The plate was incubated for 20 h at 4 °C with shaking at 300 rpm. A second washing cycle was performed, and 100 µL/well of 3,3′,5,5′-Tetramethylbenzidine (TMB) (Kem-En-Tec Diagnostics, Taastrup, Denmark, cat. no. 4380) was added and incubated for 15 min in the darkness at 20 °C with shaking at 300 rpm. The reaction was stopped by adding 100 µL/well of 1% H_2_SO_4_. The absorbance was measured at 450 nm with 650 nm as a reference using a VersaMax ELISA microplate reader (Molecular Devices, San Jose, CA, USA). A standard curve was generated using a 20 µL/well of 125 ng/mL ^1596^GPPGPPGQCDPSQCAYFASLAARPGNVKGP^1626^ standard peptide, serially diluted two-fold, using buffer alone as a reference value. A four-parametric logistic regression model was used to fit a curve. Each plate included two kit controls of a peptide-in-assay-buffer and three quality control samples comprising one human serum sample, one pig serum sample, and one synovial fluid sample. Standard curve values were accepted within a recovery percentage (RE%) < 10% and the coefficient of variance (CV%) < 15%, and the sample and control measurements were accepted with a CV% < 15%.

### 2.3. Technical Validation of the PRO-C22 ELISA

The specificity of the HRP-mAb for the C-terminal epitope was evaluated by signal inhibition. The specificity was tested using two-fold dilutions of standard peptides. Here was both a ten AA version ^1616^AARPGNVKGP^1626^ and a 30 AA version (^1596^GPPGPPGQCDPSQCAYFASLAARPGNVKGP^1626^), compared to a truncated (^1616^AARPGNVKG^1625^), and an elongated (^1616^AARPGNVKGP^1626^+A) version. Additionally, an unrelated non-sense 30 AA and a non-sense coater peptide were evaluated, along with peptides originating from the extracellular proteins, with considerable sequence similarities to the C-terminal epitope of COL22. All peptides were purchased from Genscript (Piscataway, NJ, USA).

The lower and upper limit of the measurement range (LLMR and ULMR), defined as the concentration limits of the linear range of the assay, were determined as the mean across ten independent runs. From the same ten runs, the variation was evaluated through inter- and intra-assay tests and served as a surrogate of repeatability or precision. The variation was determined, based on ten samples covering the LLMR-ULMR measurement range. The ten samples comprised three human serum samples, a two peptide-in-assay-buffer, two kit controls and three quality control samples. The intra-assay variation was calculated as the mean CV% within the plates. The inter-assay variation was based on the mean CV% between thee plates and the criteria of acceptance was CV% ≤ 20%. The analyte stability was evaluated by extending the storage of aliquoted serum samples for 2, 4, 24, or 48 h at both 4 °C and 20 °C. The RE% was calculated, relative to the control serum samples, thawed upon analysis, and accepted within 80–120%. The stability was further evaluated through the repeated freeze-thaw cycles of the serum samples: up to five rounds of freeze-thaw cycles. The freeze-thaw stability was calculated, based on the RE% relative to the samples that underwent a single thawing prior to the analysis and was accepted if the RE% was within 80–120%. The technical stability of the assay was evaluated by three runs of the assay using kit reagents incubated at 20 °C for 24 h, and the calculation of the RE% of the ten samples measured using the reagents from frozen storage. The acceptance criteria for the RE% was within 80–120%.

### 2.4. Type I and III Collagen Assessment

The assessment of type I and type III collagen was achieved by measuring the serum samples using ELISA for the detection of PRO-C1 (Nordic Bioscience A/S, Herlev, Denmark, cat. nr. 2800AF01) [49] and PRO-C3 (Nordic Bioscience A/S, Herlev, Denmark, cat. nr. 1700AF06) [50], according to the manufacturer’s instructions.

### 2.5. Patient Samples

Cohort 1 comprised serum from 11 groups of patients with different solid malignancies. Each group included 20 patients diagnosed with bladder, breast, colorectal, gastric, head and neck, lung, pancreatic, prostate, or renal cancer (n = 220). The serum from the patients with cancer was compared to the serum from sex- and age-matched self-reported healthy subjects (controls, n = 33) (Table 1). All serum samples from cancer patients were obtained from Proteogenex (Los Angeles, CA, USA), and the controls were obtained from BioIVT (Westbury, NY, USA). The sample collection was obtained after patients gave their informed consent and with the approval by the Russian Oncological Research Centre n.a. Blokhin RAMS (PG-ONC 2003/1) (Moscow, Russia) and the Western Institutional Review Board, Inc. (Puyallup, WA, USA) (WIRB^®^Protocol #20161665). All investigations were carried out in accordance with the Helsinki Declaration.

Cohort 2 included patients with pancreatic ductal adenocarcinoma (PDAC) (stages I-IV) (n = 34), and self-reported healthy subjects (n = 20). All serum samples were measured blinded before receiving information of the clinical characteristics, which included: age, sex, stage (according to the *American Joint Committee on Cancer Staging Manual*, Eighth Edition), performance status and survival time. These patient demographics (except for the survival time) are shown in (Table 2). Serum from healthy subjects was obtained from Valley BioMedical (Winchester, VA, USA).

The patient serum samples, in Cohort 2, were pre-treatment serum samples from patients with PDAC from the Danish BIOPAC study entitled “Biomarkers in patients with pancreatic cancer–can they provide new information of the disease and improve diagnosis and prognosis of the patients” (ClinicalTrials.gov ID: NCT03311776) [51].

Serum samples were obtained before the first treatment (surgery or first-line palliative chemotherapy) and processed according to the nationally approved standard operating procedures for blood (www.herlevhospital.dk/biopac, accessed on 22 November 2022). The clinical data was collected from patients prospectively but used retrospectively to evaluate the prognostic biomarker potential. The retrospective study (using serum samples from the BIOPAC study) entitled “Prognostic potential of serum biomarkers reflecting tumor fibrosis (desmoplasia) and ulceration in patients with pancreas cancer” was approved in 2016. The patients were recruited from Herlev hospital and Rigshospitalet, Denmark, during the period August 2015–September 2018. Patients were followed until May 2022 or until death. All of the patients with PDAC were histologically confirmed and were operated and/or treated with different types of chemotherapy, according to the Danish national guidelines.

The BIOPAC study was carried out, according to the Danish Regional Committee on Health Research Ethics recommendations. The BIOPAC protocol was approved by the Danish Regional Committee on Health Research Ethics (VEK ref. KA-20060113; and the retrospective protocol VEK ref. H-17039022) and the Data Protection Agency (j.nr. 2006-41-6848, 2012-58-0004, HGH-2015-027; I-Suite j. nr. 03960; and PACTIUS P-2020-834). All subjects gave written informed consent in accordance with the Declaration of Helsinki, version 8.

### 2.6. Data and Statistical Analysis

The statistical comparisons of PRO-C1, PRO-C3, and PRO-C22 levels between the healthy subjects and patients with cancer in Cohort 1 were generated using a nonparametric one-way ANOVA analysis (Kruskal–Wallis test), followed by Dunn’s multiple comparison test, comparing each solid cancer type to the healthy subjects. For the multiple comparison tests, the multiplicity-adjusted *p*-values were reported. The diagnostic accuracy of PRO-C22 was evaluated in both cohorts using a receiver operating characteristic (ROC)-curve analysis. From the sensitivity and specificity, the ROC curves were generated and the area under the ROC curves (AUROCs) were calculated.

Spearman’s correlation analysis was used to correlate the PRO-C22 levels with the levels of PRO-C1 and PRO-C3, respectively. This was achieved by plotting PRO-C22 as a function of the interstitial matrix biomarkers, respectively, based on the relative measured values. The correlation coefficient (r_s_) and *p*-value were interpreted according to the Rule of Thumb for Interpreting the Size of a Correlation Coefficient [52].

To investigate the prognostic value of PRO-C22 and PRO-C3, the biomarker levels were dichotomized, based on tertiles (T1, T2, and T3) and the patients were divided into “low” (e.g., PRO-C22_low_ = T1 + T2) and “high” (e.g., PRO-C22_high_ = T3) subgroups. From these groups, Kaplan–Meier survival plots were created to assess the association between OS and the serum levels of PRO-C22 and PRO-C3 in patients with PDAC (Cohort 2).

The prognostic value was also evaluated using a univariate Cox proportional-hazard regression model to assess the hazard ratio of mortality for PRO-C22, PRO-C3, age, stage, and sex, respectively. Multivariate Cox proportional-hazard regression models were used to evaluate if the associations between OS and the biomarker levels were independent of metastasis by adjusting for stage IV PDAC. This was achieved by calculating the hazard ratios (HRs) within a 95% confidence interval (95% Cl) for short OS using low vs. high biomarker levels (T1 + T2 vs. T3).

To further evaluate the prognostic performance and a potential additive value by combining PRO-C3 and PRO-C22, Harrell’s C-statistics were applied. This was supported by the classification and a regression tree (CART) analysis.

For all statistical analyses, a *p* < 0.05 was considered statistically significant. The statistical analyses and graphs were made using GraphPad Prism (version 9.4.0 for Windows, GraphPad Software, San Diego, CA, USA) and MedCalc Statistical Software (version 19.3, MedCalc Software bvba, Ostend, Belgium.

## 3. Results

### 3.1. The PRO-C22 ELISA Assay Was Validated as a Specific and Technically Robust Biomarker

**Figure 1:**
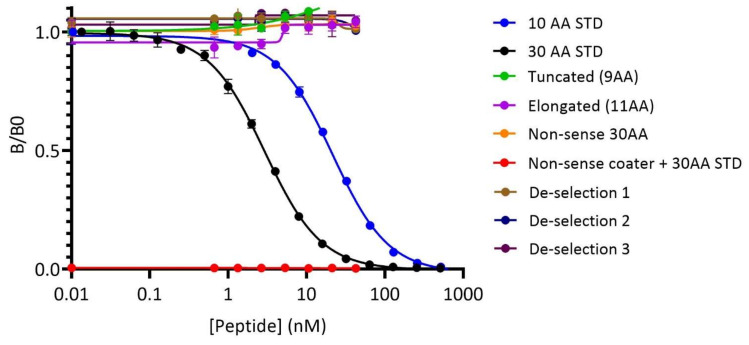
The PRO-C22 ELISA was specific for the target epitope. Dose-dependent signal inhibition of the interaction observed between the biotinylated coating peptide (Biotin-^1616^AARPGNVKGP^1626^) and the horseradish peroxidase labeled monoclonal antibody (HRP-mAb) by the assay-specific ten and 30 amino acid (AA) standard peptides (^1616^AARPGNVKGP^1626^ and ^1596^GPPGPPGQCDPSQCAYFASLAARPGNVKGP^1626^, respectively). Lack of inhibition was observed by the truncated peptide (^1616^AARPGNVKG^1625^), elongated peptide (^1616^AARPGNVKGP^1626^+A), or the non-sense 30 AA peptide. The non-sense coating peptide resulted in no signal generation. Three de-selection peptides corresponding to sequences from the extracellular matrix proteins and with sequence similarities to the PRO-C22 epitope showed no effect. B/B0 is the ratio between B (the signal intensity when a sample or peptide is present) and B0 (the maximum signal intensity generated when no sample or peptide is present).

Following the mAb generation, the supernatant was collected and the mAb was purified. The supernatant contained 42.6 µg/mL mAb and resulted in a concentration of 3.5 mg/mL purified desalted mAb. Through various optimizing steps, the best competitive setting of the PRO-C22 ELISA assay was defined. This included the identification of the appropriate buffer, incubation time, temperature, concentrations of the antibody and peptides, and involved the horseradish peroxidase labeling of the mAb (HRP-mAb). The assay used a standard peptide (calibrator) that, by outcompeting the binding of the HRP-mAb to the biotinylated peptide (coater), was used to quantify the circulating COL22 in the serum. The specificity was tested using a truncated and elongated version of the peptide that did not show any competition for the HRP-mAb. A non-sense coater and a non-sense 30 AA standard peptide were evaluated for the ability to interfere with the competition as well, none of them showed any effect. In contrast, both the ten and the 30 AA sequence standard peptide successfully inhibited the interaction dose, dependently (Figure 1).

The linear measurement range and the lower limit of quantification (LLOQ) were 1.0–53.0 ng/mL and 1.95 ng/mL, respectively. The precision of the assay was determined through intra- and inter-variation tests. Both the intra- and inter-variations were 4%. The minimum required dilution of the serum was evaluated by the linearity of the healthy serum samples two-fold diluted in the assay. This determined 1:2 as the minimum required dilution and limited the maximum dilution to 1:4 of the healthy serum samples. This was based on a mean RE% of 104%. The accuracy was determined by spiking the (30AA) PRO-C22 standard peptide into human serum samples of a known concentration, as well as spiking the human serum samples of known concentrations into new human serum samples. The spiking recovery was calculated as the measured PRO-C22, relative to the expected concentration. The mean RE% of the peptide in serum was 105% and the mean RE% of the serum in serum was 104%. The interference was tested by spiking the low/high volumes of biotin (5/100 ng/mL), lipids (1.5/5.0 mg/mL), and hemoglobin (0.5/2.5 mg/mL), into a serum sample of a known PRO-C22 concentration. The analyte recoveries for the interference of biotin, lipids, and hemoglobin ranged from 85% to 113%.

The stability was evaluated regarding both the analyte and kit reagent stability. The mean RE% of the three serum samples that underwent five freeze-thaw cycles was 103%. The serum samples incubated up to 48 h at 4 °C had a RE% of 107%, and 106% for samples incubated up to 48 h at 20 °C. The kit reagents (buffer, biotinylated-peptide and HRP-mAb) were evaluated after 24 h of incubation at 20 °C. The stability of the reagents was evaluated by measuring the same ten samples used for intra- and inter-variation and calculating the RE%. The ten samples measured in three individual runs, showed a mean RE% of 94% (Table 3). 

### 3.2. PRO-C22 Was Significantly Elevated in the Serum from Patients with Solid Cancers

**Figure 2:**
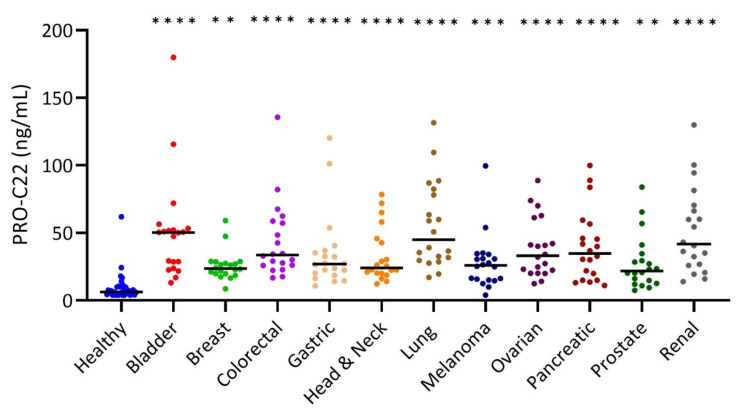
PRO-C22 was significantly elevated in the serum from patients with solid tumors. Quantification of PRO-C22 in the serum of healthy subjects (n = 33) and bladder cancer (n = 20), breast cancer (n = 20), colorectal cancer (n = 20), gastric cancer (n = 20), head and neck cancer (n = 20), lung cancer (n = 20), melanoma cancer (n = 20), ovarian cancer (n = 20), pancreatic cancer (n = 20), prostate cancer (n = 20), and renal cancer (n = 20). PRO-C22 levels are presented individually as scatter plots with black bars marking the median. Samples measuring below the LLOQ were given the LLOQ value (of 3.9 ng/mL) determined in the validation of PRO-C22. Differences in the PRO-C22 levels between the cancer-types and the healthy subjects were calculated using a Kruskal–Wallis test followed by multiple comparisons to the controls with Dunn’s test. **** indicates *p* < 0.0001, *** *p* < 0.001, ** *p* < 0.01.

In Cohort 1, PRO-C22 was significantly elevated in all groups of patients with cancer (bladder, colorectal, gastric, head and neck, lung, ovarian, pancreatic, and renal cancer (*p* < 0.0001), melanoma (*p* < 0.001), breast, and prostate cancer (*p* < 0.01)) compared to the healthy subjects (Figure 2). The median of PRO-C22 ranges from 21.8 to 50.3 ng/mL in the patients with cancer, compared to 6.2 ng/mL in the healthy subjects (Figure 2). There was moreover a pronounced inter-subject variation of PRO-C22 in patients with cancer, compared to healthy subjects. Furthermore, the AUROC varied from 0.89–0.98 (*p* < 0.0001) (Table 4).

### 3.3. Interstitial Matrix Biomarkers PRO-C1 and PRO-C3 in Solid Tumor Types

**Figure 3:**
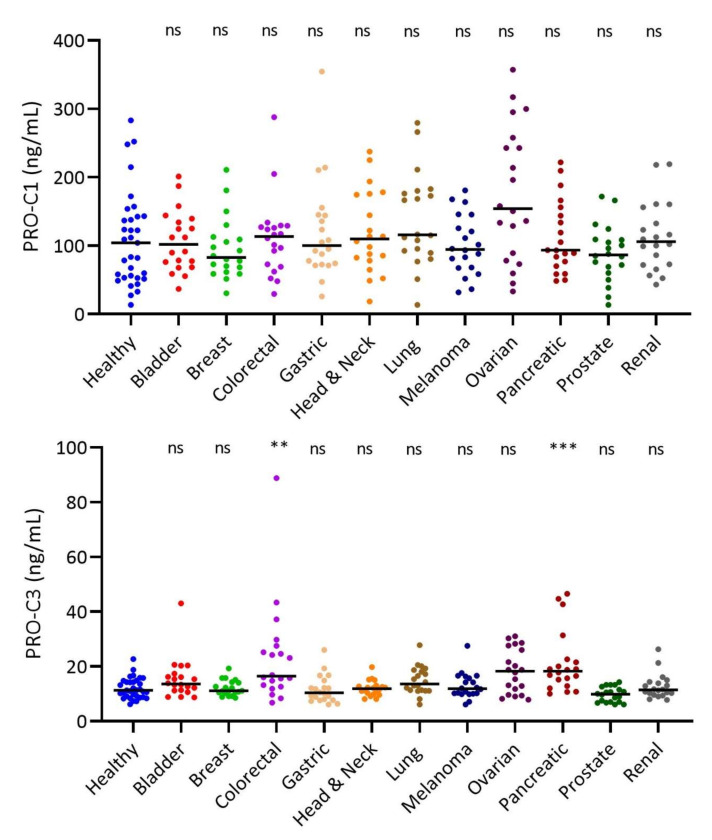
PRO-C1 and PRO-C3 are different from PRO-C22. Quantification of PRO-C1 (top) and PRO-C3 (bottom) in the serum of healthy subjects (n = 33) and bladder cancer (n = 20), breast cancer (n = 20), colorectal cancer (n = 20), gastric cancer (n = 20), head and neck cancer (n = 20), lung cancer (n = 20), melanoma cancer (n = 20), ovarian cancer (n = 20), pancreatic cancer (n = 20), prostate cancer (n = 20), and renal cancer (n = 20). PRO-C1 and PRO-C3 levels are presented individually as scatter plots with black bars marking the median. Samples measuring below the LLOQ were given the LLOQ value. Significant differences in the biomarker levels (PRO-C1 and PRO-C3) between the cancer-types and the healthy subjects were calculated using a Kruskal–Wallis test followed by multiple comparisons to the controls with Dunn’s test. *** *p* < 0.001, ** *p* < 0.01, ns indicates *p* > 0.05.

The major fibrillar collagens, COL1 and COL3, have shown their importance in cancer progression. Therefore, we investigated the formation of COL1 and COL3 by measuring PRO-C1 and PRO-C3 in the serum from the 11 groups of patients with cancer. In contrast to PRO-C22, there was no significant difference in the PRO-C1 levels between healthy subjects and patients with solid tumor types. PRO-C3 was only significantly elevated in patients with colorectal and pancreatic cancer (*p* = 0.0052 and *p* = 0.0009, respectively) (Figure 3). The associations between PRO-C22 and PRO-C1 or PRO-C3 showed low correlation coefficients with r_s_ = 0.20 (*p* < 0.0001), and r_s_ = 0.38 (*p* < 0.0001), respectively (Supplementary Figure S1).

### 3.4. PRO-C22 and PRO-C3 Were Significantly Elevated in Patients with PDAC (Cohort 2)

**Figure 4:**
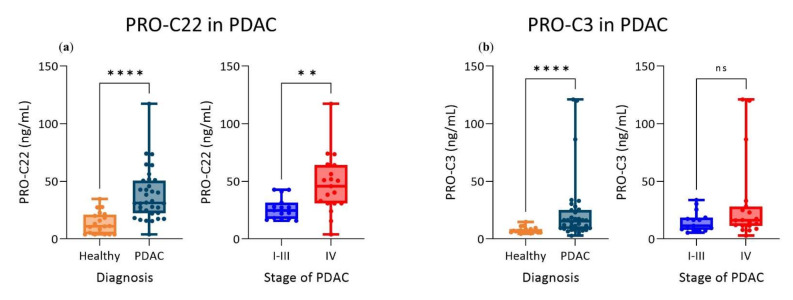
PRO-C22 and PRO-C3 were significantly elevated in patients with pancreatic ductal adenocarcinoma (PDAC). (**a**) Levels of PRO-C22 were significantly elevated in patients with PDAC (n = 34), compared to healthy subjects (n = 20). PRO-C22 was increased in patients with metastatic PDAC (stage IV, n = 19), compared to non-metastatic PDAC (stage I–III, n = 15). (**b**) Levels of PRO-C3 were significantly elevated in PDAC patients (n = 34), compared to healthy subjects (n = 20). Comparisons were based on nonparametric *t*-tests (Mann–Whitney test). **** indicates *p* < 0.0001, ** indicates *p* < 0.01, ns indicates a *p* > 0.05.

In Cohort 2, PRO-C22 was significantly elevated in serum from patients with PDAC, compared to healthy subjects (*p* < 0.0001). Moreover, PRO-C22 could differentiate between patients with metastatic PDAC (stage IV, n = 19) and patients with non-metastatic PDAC (stages I–III PDAC, n = 15) (*p* = 0.0031) (Figure 4a). The linear regression analysis additionally showed that the increasing levels of PRO-C22 were associated with the increased stage (slope = 20.8 and *p* = 0.0051) (data not included). PRO-C3 was assessed in the same cohort and was similarly significantly elevated in the serum from patients with PDAC, compared to healthy subjects (*p* < 0.0001), however no significant difference was observed between metastatic PDAC (stage IV) and non-metastatic PDAC (stages I–III), according to the PRO-C3 levels (*p* = 0.2821) (Figure 4b).

### 3.5. Prognostic Perspective of PRO-C22 and PRO-C3 in Patients with PDAC (Cohort 2)

**Figure 5:**
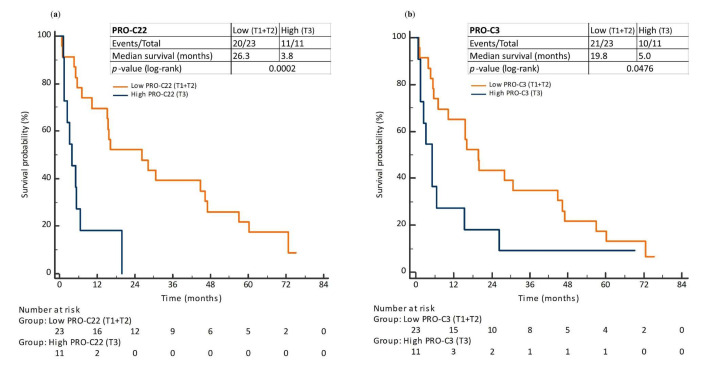
High levels of PRO-C22 and PRO-C3 are associated with a short overall survival (OS) in patients with PDAC. Kaplan–Meier survival plots showing the association between the OS and PRO-C22 (**a**) and PRO-C3 (**b**) levels in patients with PDAC. Orange lines shows the low biomarker levels (tertiles 1 + 2 = T1 + T2), blue lines show the high biomarker levels (tertile 3 = T3).

To investigate the prognostic value of PRO-C22 and PRO-C3, patients in Cohort 2, their biomarker levels were dichotomized into “low” (T1 + T2) and “high” (T3) subgroups. The Kaplan–the Meier survival analysis showed a difference of 22.5 months in median OS time between patients in the PRO-C22_low_ and PRO-C22_high_ subgroups (median OS time of PRO-C22_high_: 3.8 months vs. PRO-C22_low_: 26.3 months, log-rank *p* = 0.0002) (Figure 5a). The Kaplan–Meier survival analysis based on the PRO-C3 levels showed that the difference in median OS time between the PRO-C3_low_ and PRO-C3_high_ subgroups was 14.8 months (median OS time of PRO-C3_high_: 5.0 months vs. PRO-C3_low_: 19.8 months, log-rank *p* = 0.0476) (Figure 5b).

A univariate Cox proportional-hazard regression analysis showed that high levels of PRO-C22 (T3) were significantly associated with a short OS (HR = 4.52, 95% CI 1.90–10.7, *p* = 0.0006), compared to low levels of PRO-C22 (T1 + T2). Similar analysis was used to evaluate the high, compared to the low levels of PRO-C3 (T3 vs. T1 + T2) and showed that a high PRO-C3 was non-significantly associated with a short OS (HR = 2.15, 95% CI 0.99–4.68, *p* = 0.0527). (Table 5).

As the stage of the disease was significantly associated with OS (Table 5) this variable was incorporated in a multivariate Cox proportional-hazard regression analysis, to investigate whether the prognostic value of PRO-C22 was independent of metastasis (stage IV PDAC). By using the same dichotomization of the PRO-C22_low_ and PRO-C22_high_ subgroups (T3 vs. T1+T2) the multivariate model, adjusted for metastatic PDAC (stage IV of disease), showed that patients with high levels of PRO-C22 still had a significantly increased risk of a poor survival outcome (HR = 3.12, 95% CI 1.24–7.85, *p* = 0.0159), independent of the stage (Table 6).

Similar analyses were used to evaluate the prognostic potential of PRO-C3 in the same group with PDAC (Cohort 2). A multivariate Cox proportional-hazard regression analysis showed that PRO-C3 also was prognostic for a short OS with a HR of 2.91 (95% CI of 1.26–6.71, *p* = 0.0121), independent of metastasis (Table 7).

To investigate if PRO-C22 was prognostically independent of PRO-C3, we included both biomarkers in a multivariate Cox proportional-hazard regression analysis. The model showed that a high PRO-C22 was significantly associated with a poor survival outcome, independent of the high PRO-C3 levels (HR = 4.27, 95% CI 1.24–10.4, *p* = 0.0013) (Table 8).

Evaluation of the prognostic potential of PRO-C22 and PRO-C3 (individually and combined) was investigated using Harrell’s C-index. The C-index was 0.61 (95% CI 0.52–0.70) and 0.64 (95% CI 0.57–0.72) for PRO-C3 and PRO-C22, respectively. Combining of the biomarkers increased the C-index slightly to 0.68 (in a 95% CI 0.60–0.76), and this may indicate an additive value of PRO-C22 and PRO-C3.

**Figure 6:**
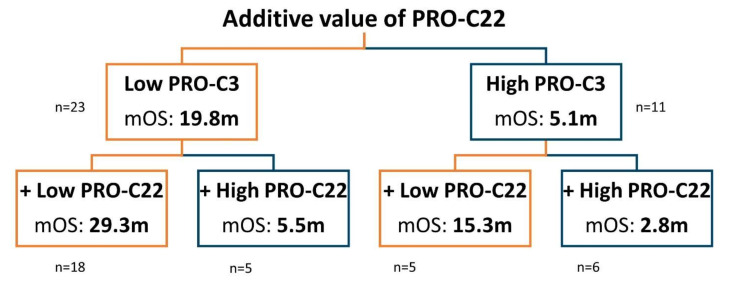
Classification and regression tree (CART) analysis to explore the additive value of PRO-C22 to PRO-C3. Dichotomization of the patients into low PRO-C3 levels (tertiles 1 + 2 = T1 + T2, orange) and high PRO-C3 levels (tertile 3 = T3, blue). The following branches of the tree represent the dichotomization of patients that additionally have low (T1 + T2, orange) or high (T3, blue) PRO-C22 levels. Median overall survival time (mOS) in months (m) and the number of patients (n) is listed in each group.

A classification and regression tree (CART) analysis was used to evaluate the potential additive value of PRO-C22 to PRO-C3. This resulted in four subgroups of patients: PRO-C3_low_ + PRO-C22_low_ (LL), PRO-C3_low_ + PRO-C22_high_ (LH), PRO-C3_high_ + PRO-C22_low_ (HL), and PRO-C3_high_ + PRO-C22_high_ (HH). The median OS time was 29.3 months for the patients in the LL group (n = 18), 5.5 months in the LH group (n = 5), 15.3 months in the HL group (n = 5), and 2.8 months in the HH group (n = 6) (Figure 6).

## 4. Discussion

In this study, we developed a novel ELISA assay (PRO-C22) to measure COL22 in serum. In addition, we studied the diagnostic and prognostic value of this biomarker, and the associations with other tumor fibrosis biomarkers. PRO-C22, measuring a minor FACIT collagen, showed a superior diagnostic property, compared to PRO-C1 and PRO-C3, measuring major collagen fragments. Furthermore, PRO-C22 provided additive information to the well-known tumor fibrosis biomarker PRO-C3.

The ECM-derived molecules are released into circulation as part of the alterations of the TME and cancer progression [53]. PRO-C22 was therefore evaluated in the serum from patients with different types of solid tumors. Despite the relatively pronounced inter-patient variation, the AUROCs ranged from 0.89 to 0.98 depending on the cancer type. PRO-C22 was moreover significantly elevated across all cancer types in Cohort 1, indicating that PRO-C22 could be an efficient tool to identify cancer patients. The diagnostic accuracy of PRO-C22 is noteworthy when compared to the levels of PRO-C1 and PRO-C3, which quantify abundant fibrillar COL1 and COL3 collagens. These collagens are often used histologically to assess tumor fibrosis in the tissue using sirius red or picro-sirius red staining [54,55,56].

The lack of a general discrepancy in (PRO-C1 and PRO-C3) biomarker levels in the serum from healthy subjects and cancer patients in Cohort 1 might be caused by the already high systemic levels, since COL1 and COL3 are the most abundant collagens [57]. PRO-C3 was significantly elevated in the serum from patients with colorectal and pancreatic cancer, malignancies, known to have a deposition of fibrillar collagens [58,59,60], and a high PRO-C3 has previously been associated with mortality in cancer patients with a metastatic disease [19,61,62].

As both fibrillar collagens and FACITs have been shown to play a role in the cancer progression, we investigated if the FACIT biomarker PRO-C22 could provide additional prognostic value to PRO-C3. In Cohort 2, comprising patients with PDAC, both PRO-C22 and PRO-C3 were significantly elevated in patients with cancer, confirming our initial observations from Cohort 1. PRO-C22 was significantly elevated in patients with stage IV PDAC, compared to the lower stages, suggesting that PRO-C22 might be associated with tumor burden. We also demonstrated that patients with PDAC, with high levels of circulating COL22 had a 4.5-fold increased risk of mortality, and high levels of both PRO-C22 and PRO-C3 were prognostic for a poor OS, independent of the disease stage.

Interestingly, based on the C-index and CART analysis, PRO-C22 added a prognostic value to PRO-C3, suggesting that the biomarkers are complementary and may reflect two separate biological processes. While this study is the first to associate COL22 with the outcome in PDAC, the results are in line with what has been shown for COL22 in squamous cell carcinoma of the head and neck, where the mRNA expression of COL22A1 was significantly increased and associated with an earlier disease recurrence and thus a shorter disease-free survival [47].

While induction of PRO-C3 has been linked to cancer-associated fibroblasts and significantly induced by transforming the growth factor-beta (TGF-β) [61], a direct link between PRO-C22 and tumor fibrosis, has not yet been demonstrated. Interestingly, the COL22A1 expression may be an early response marker to TGF-β, based on the data from human ex-vivo and in vitro models [63]. The increased expression of COL22A1 along with TGF-β1 and TGF-β2, has been seen in response to the estradiol induced dermal fibrosis. This association could mechanistically be blocked using an estrogen receptor α- or TGF-β receptor inhibitor (SB-431542) [64]. Thus, it seems that PRO-C22 and PRO-C3 reflect different aspects of the TGF-β signaling, as well as tumor fibrosis. Other known aspects of cancer-associated ECM remodeling include the increased proteolytic activity causing the degradation of the basement membrane (BM) components. This involves FACITs and COL19, as an example, has been proposed to act as a “first-line” of defense before the breakdown of the BM constituents in breast cancer [65]. COL22 like COL19, is associated with the BM [33,35,66,67,68,69,70]. Based on this similar spatial distribution feature, one might speculate that COL22 (measured by PRO-C22) could reflect similar early aspects of the TME alterations.

Although most FACITs are made up of three identical alpha chains, they are structurally very diverse [25,71]. Based on the structural features, COL22 may resemble COL16 and COL19 the most. They all hold a thrombospondin domain followed by more than four collagenous domains interspersed by non-collagenous domains [25,71], whereas COL12, COL14, COL20, and COL21 only hold two triple-helical collagenous domains that are interrupted by a single non-triple helical domain. Different from COL16 and COL19, COL22 holds a von Willebrand Factor A domain, a feature shared with COL12, COL14, COL20, and COL21. Only COL12, COL14, and COL20 have several fibronectin type III domains. Still, the FACITs are most often described collectively and as molecular bridges between the ECM fibrils through their C-terminal domain [72]. Since the FACITs are associated with fibrillar assembly, it could be speculated that COL22 is produced in advance of fibrils causing an increased amount of C-terminal COL22, as measured using PRO-C22. Interestingly, a recent study showed that COL12 could alter the organization of COL1, fostering a more pro-invasive microenvironment [39]. Therefore, the property of FACITs in the ECM organization should be further explored in the context of cancer.

There are several limitations to the present study. The retrospective nature of the study and the small sample size could introduce biases and therefore it is necessary to validate these findings in independent prospective studies. Moreover, an optimal cut-point needs to be further explored [73]. Despite that the PRO-C3 data is similar in other PDAC cohorts, it is possible that the selection bias may also have occurred in Cohort 2 as only patients in good performance for operation or palliative chemotherapy are included in the BIOPAC study [62].

As increased inflammation affects the remodeling of the ECM, attention should also be paid to the evaluation of the biomarker potential in chronic disorders. This would especially underline the applicability of the biomarker in a clinical setting, if PRO-C22 could discriminate patients with solid cancer from patients with inflammation in similar tissues, such as pancreatitis or inflammatory bowel disease. Additionally, it could be interesting to evaluate if a pharmacodynamic effect of PRO-C22 could be found in patients receiving anti-stromal treatments. Based on these preliminary data we think that FACITs are an important field to take into consideration when studying both cancer biology and anti-cancer treatments and we see a potential in applying PRO-C22 in future studies within this field.

## 5. Conclusions

The major findings in this study included the development and technical validation of a novel non-invasive serological biomarker, PRO-C22. The PRO-C22 assay quantified the C-terminal epitope of COL22 released to circulation from patients with various solid cancers. PRO-C22 demonstrated diagnostic potential and high levels were prognostic for a short OS in patients with PDAC, where it complimented the tumor fibrosis biomarker PROC-3. This indicates that PRO-C3 and PRO-C22 reflect different aspects of tumor fibrosis and emphasizes the diversity in biological properties, and functions of the various collagens.

## Figures and Tables

**Table 1:** Demographics of Cohort 1, patients with different solid cancers.

	Healthy Subjects (n = 33)	Patients with Cancer (n = 220)
Healthy	33 (100%)	
**Diagnosis**		
Bladder cancer		20 (9%)
Breast cancer		20 (9%)
Colorectal cancer		20 (9%)
Gastric cancer		20 (9%)
Head and neck cancer		20 (9%)
Lung cancer		20 (9%)
Melanoma		20 (9%)
Ovarian cancer		20 (9%)
Pancreatic cancer		20 (9%)
Prostate cancer		20 (9%)
Renal cancer		20 (9%)
**Stage**		
I		7 (3%)
II		46 (21%)
III		93 (42%)
IV		74 (34%)
**Age (years)**		
Mean (SD)	58 (6)	59 (11)
Median (Min; Max)	57 (49; 69)	61 (30; 87)
Missing		1 (0.5%)
**Sex**		
Male	21 (64%)	119 (54%)
Female	12 (36%)	101 (46%)

**Table 2:** Demographics of Cohort 2, patients with pancreatic ductal adenocarcinoma (PDAC).

	Healthy Subjects (n = 20)	Patients with PDAC (n = 34)
**Stage**		
I		1 (3%)
II		7 (20%)
III		7 (20%)
IV		19 (56%)
Missing	20 (100%)	
**Age (years)**		
Median (Min; Max)	58 (45; 72)	69 (52; 79)
**Sex**		
Male	10 (50%)	18 (53%)
Female	10 (50%)	16 (47%)
**Performance status**		
0		14 (41%)
1		12 (35%)
2		3 (9%)
Unknown		5 (15%)

**Table 3:** Technical validation data for PRO-C22.

Test	Result
Measurement range	1–53 ng/mL
Lower limit of quantification (LLOQ) in serum	2 ng/mL
Dilution recovery of serum (1:2 to 1:4)	104%
Spiking recovery of peptide in serum	105%
Spiking recovery of serum in serum	97%
Biotin interference recovery, low/high concentration	99%/85%
Lipid interference recovery, low/high concentration	105%/102%
Hemoglobin recovery, low/high concentration	101%/113%
Inter-assay variation	4%
Intra-assay variation	4%
Analyte stability (48 h 4 °C/48 h 20 °C)	107%/106%
Freeze-thaw stability up to five cycles	103%
Kit stability (24 h 20 °C)	94%

**Table 4:** Area under the receiver operating characteristic (AUROC) for evaluating the separability of patients with solid tumors from healthy subjects using PRO-C22. The 95% confidence interval (95% CI) are given in parenthesis.

Solid Tumor Type (n = 20)	AUROC (95% CI)	Sensitivity% (95% CI)	Specificity% (95% CI)
**Bladder**	0.96 (0.87–1.00)	95 (75.1–99.9)	91 (75.7–98.1)
**Breast**	0.94 (0.83–0.98)	95 (75.1–99.9)	88 (71.8–96.6)
**Colorectal**	0.98 (0.88–1.00)	100 (83.2–100.0)	91 (75.7–98.1)
**Head and Neck**	0.95 (0.85–0.99)	100 (83.2–100.0)	85 (68.1–94.9)
**Gastric**	0.94 (0.84–0.99)	95 (75.1–99.9)	88 (71.8–96.6)
**Lung**	0.98 (0.89–1.00)	100 (83.2–100.0)	91 (75.7–98.1)
**Melanoma**	0.89 (0.77–0.96)	90 (68.3–98.8)	85 (68.1–94.9)
**Ovarian**	0.96 (0.86–0.99)	100 (83.2–100.0)	85 (68.1–94.9)
**Pancreatic**	0.94 (0.84–0.99)	100 (83.2–100.0)	82 (64.5–93.0)
**Prostate**	0.90 (0.79–0.96)	90 (68.3–98.8)	82 (64.5–93.0)
**Renal**	0.97 (0.88–1.00)	100 (83.2–100.0)	85 (68.1–94.9)

**Table 5:** Univariate analysis of survival using PRO-C22, PRO-C3 and the clinical variables associated with a poor prognosis. Biomarkers were dichotomized according to high versus (vs.) low levels, based on the tertiles (T3 vs. T1 + T2). The hazard ratio (HR), as well as a 95% confidence interval (CI) and a *p*-value, is listed for each analysis.

Variable	Cut-Point	HR	95% CI	*p*
PRO-C22	T3 vs. T1 + T2	4.52	1.90 to 10.7	0.0006
PRO-C3	T3 vs. T1 + T2	2.15	0.99 to 4.68	0.0527
Stage	Stage IV vs. Stage I–III	3.26	1.53 to 6.96	0.0022
Age	Continuous	1.02	0.97 to 1.06	0.4805
Sex	Female vs. Male	0.67	0.32 to 1.41	0.2957

**Table 6:** Multivariate analysis of survival using PRO-C22 (adjusted for the co-variate stage IV of the disease). PRO-C22 was dichotomized according to high versus (vs.) low levels, based on the tertiles (T3 vs. T1 + T2). The hazard ratio (HR), 95% confidence interval (CI), and a *p*-value are listed.

PRO-C22	Cut-Point	HR	95% CI	*p*
Co-variate				
Stage (IV vs. I–III)	T3 vs. T1 + T2	3.12	1.24 to 7.85	0.0159

**Table 7:** Multivariate analysis of survival using PRO-C3 (adjusted for the co-variate stage IV of the disease). PRO-C3 was dichotomized according to high versus (vs.) low levels, based on the tertiles (T3 vs. T1 + T2). The hazard ratio (HR), 95% confidence interval (CI), and a *p*-value are listed.

PRO-C3	Cut-Point	HR	95% CI	*p*
Co-variate				
Stage (IV vs. I–III)	T3 vs. T1 + T2	2.91	1.26 to 6.71	0.0121

**Table 8:** Multivariate analysis of survival using PRO-C22 (adjusted for the co-variate high levels of PRO-C3 (T3)). PRO-C22 was dichotomized according to high versus (vs.) low levels, based on the tertiles (T3 vs. T1 + T2). The hazard ratio (HR), 95% confidence interval (CI), and a *p*-value are listed.

PRO-C22	Cut-Point	HR	95% CI	*p*
Co-variate				
PRO-C3 (T3 vs. T1 + T2)	T3 vs. T1 + T2	4.27	1.76 to 10.4	0.0013

## Data Availability

The data presented in this study can be available upon request and obtained from the corresponding author.

## Supplementary Materials

The following supporting information can be downloaded at: https://www.mdpi.com/article/10.3390/cells11233763/s1, Figure S1: Biomarker correlations; Table S1: Ability of PRO-C22 to separate patients with pancreatic ductal adenocarcinoma (PDAC) from healthy subjects; Table S2: Ability of PRO-C3 to separate patients with pancreatic ductal adenocarcinoma (PDAC) from healthy subjects.

## Abbreviations

AA (amino acid), AUROC (area under the receiver operating characteristic), BM (basement membrane), BSA (bovine serum albumin), CAFs (cancer-associated fibroblasts), COL1 (type I collagen), COL3 (type III collagen), COL9 (type IX collagen), COL12 (type XII collagen), COL14 (type XIV collagen), COL16 (type IX collagen), COL19 (type XIX collagen), COL20 (type XX collagen), COL22 (type XXII collagen), C-Terminal (carboxyl-terminus of a peptide sequence), CV% (coefficient of variance), ECM (extracellular matrix), ELISA (enzyme-linked immunosorbent assay), FACITs (fibril-associated collagens with interrupted triple helices), HRP (horseradish peroxidase), HRP-mAb (horseradish peroxidase-labeled monoclonal antibody), IM (interstitial matrix), KLH (keyhole limpet hemocyanin), mAb (monoclonal antibody), NaCl (sodium chloride), nm (nano metre), OS (overall survival), PDAC (pancreatic ductal adenocarcinoma), PRO-C1 (enzyme-linked immunosorbent assay that can quantify the formation of type I collagen), PRO-C3 (enzyme-linked immunosorbent assay that can quantify the formation of type III collagen), PRO-C22 (enzyme-linked immunosorbent serum assay that targets the C-terminal epitope of type XXII collagen), RE% (recovery percentage), ROC (receiver operating characteristic), rpm (rounds per minute), TBS (Tris buffered saline), TGF-β (transforming growth factor-beta), TMB (3,3′,5,5′-Tetramethylbenzidine), TME (tumor microenvironment), TSP(thrombospondin), VWA (von Willebrand factor A).
