# Supplementary material for: Type XXII Collagen Complements Fibrillar Collagens in the Serological Assessment of Tumor Fibrosis and the Outcome in Pancreatic Cancer
Source: Cells. 2022 Nov 24;11(23):3763. doi: 10.3390/cells11233763 (PMC9738409; doi:10.3390/cells11233763)
Supplement: Supplementary file 1 [file cells-11-03763-s001.zip › cells-1973327-supplementary.pdf]

cells MDPI

Article

Type XXII Collagen Complements Fibrillar Collagens in the Serological Assessment of Tumor Fibrosis and the Outcome in Pancreatic Cancer

Emilie A. Madsen [1,2,*], Jeppe Thorlacius-Ussing [1], Neel I. Nissen [1], Christina Jensen [1], Inna M. Chen [3], Julia S. Johansen [3,4,5], Hadi M. H. Diab [6], Lars N. Jørgensen [5,6], Carsten P. Hansen [7], Morten A. Karsdal [1] and Nicholas Willumsen [1]

[1] Biomarkers and Research, Nordic Bioscience A/S, Herlev Hovedgade 207, 2730 Herlev, Denmark
[2] Department of Biomedical Sciences, University of Copenhagen, 2200 Copenhagen, Denmark
[3] Department of Oncology, Copenhagen University Hospital—Herlev and Gentofte, 2730 Herlev, Denmark
[4] Department of Medicine, Copenhagen University Hospital—Herlev and Gentofte, 2730 Herlev, Denmark
[5] Department of Clinical Medicine, Faculty of Health and Medical Sciences, University of Copenhagen, 2200 Copenhagen, Denmark
[6] Digestive Disease Center, Bispebjerg Hospital, University of Copenhagen, 2400 Copenhagen, Denmark
[7] Department of Surgery, Rigshospitalet, University of Copenhagen, 2100 Copenhagen, Denmark
* Correspondence: eam@nordicbio.com; Tel.: +45-44525252

Supplementary Data

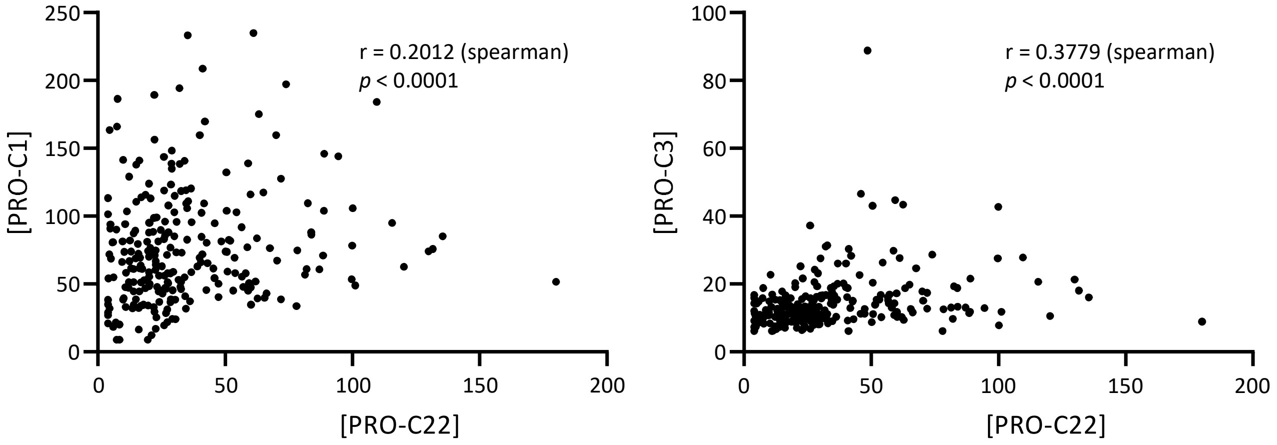

Supplementary Figure S1. Biomarker correlations. Correlation between PRO-C22 and PRO-C1 (left) and PRO-C3 (right). Brackets indicate the concentration of the given biomarker, r is the calculated spearman's correlation coefficient, p<0.05 is considered significant.

Supplementary Table S1. Ability of PRO-C22 to separate patients with pancreatic ductal adenocarci-noma (PDAC) from healthy subjects.

PRO-C22	AUROC (95% CI)	Sensitivity % (95% CI)	Specificity % (95% CI)
**PDAC (n=34)**	0.87 (0.75 to 0.95)	79 (62.1 - 91.3)	80 (56.3 - 94.3)

Supplementary Table S2. Ability of PRO-C3 to separate patients with pancreatic ductal adenocarci-noma (PDAC) from healthy subjects.

PRO-C3	AUROC (95% CI)	Sensitivity % (95% CI)	Specificity % (95% CI)
**PDAC (n=34)**	0.85 (0.73 to 0.94)	68 (49.5 - 82.6)	90 (68.3 - 98.8)
